# Efficient Nuclear Transport of Structurally Disturbed Cargo: Mutations in a Cargo Protein Switch Its Cognate Karyopherin

**DOI:** 10.1371/journal.pone.0016846

**Published:** 2011-02-09

**Authors:** Susana R. Chaves, Jonathan S. Rosenblum

**Affiliations:** 1 Centre of Molecular and Environmental Biology, Department of Biology, University of Minho, Campus de Gualtar, Braga, Portugal; 2 Howard Hughes Medical Institute, Laboratory of Cell Biology, The Rockefeller University, New York, New York, United States of America; University of Medicine and Dentistry of New Jersey, United States of America

## Abstract

The Karyopherin (Kap) family of nuclear transport receptors enables trafficking of proteins to and from the nucleus in a precise, regulated manner. Individual members function in overlapping pathways, while simultaneously being very specific for their main cargoes. The details of this apparent contradiction and rules governing pathway preference remain to be further elucidated. *S. cerevisiae* Lhp1 is an abundant protein that functions as an RNA chaperone in a variety of biologically important processes. It localizes almost exclusively to the nucleus and is imported by Kap108. We show that mutation of 3 of the 275 residues in Lhp1 alters its import pathway to a Kap121-dependent process. This mutant does not retain wild-type function and is bound by several chaperones. We propose that Kap121 also acts as a chaperone, one that can act as a genetic buffer by transporting mutated proteins to the nucleus.

## Introduction

The first step in the majority of nuclear import/export pathways is the recognition of targeting signals by soluble transport receptors (Karyopherins, or Kaps). The Kapα/β heterodimer, the best characterized carrier, transports proteins containing short stretches of basic amino acid residues, the so-called classical nuclear localization sequences (NLSs) [1]. Karyopherins other than the Kapα/β heterodimer are most often found to recognize large NLSs. Each Kap binds to different localization sequences, and thus the affinity of the Kap/signal interaction is a critical factor in determining transport efficiency and selectivity. Despite the recent advances in the field, this step is still poorly understood and the binding specificities for all but a few Kaps remain largely unknown. This is the case for the *S. cerevisiae* Kap121, a Kap for which many substrates have been identified, since the NLSs recognized by Kap121 do not share significant amino acid identity. It could be that Kap121 has more than one cargo binding site. It is also possible that Kap121 (as well as the other Kaps) recognizes structural rather than sequence elements in its cargoes. In support of this, mutation of a number of residues in Kap121 NLSs does not always result in a readily observed phenotype [2], suggesting that binding to the Kap may not be restricted to a few specific residues. It remains to be determined whether this is the case for the majority of large NLSs. We thus set out to characterize the Lhp1 NLS, which consists of 112 residues and is targeted to the nucleus in a Kap108-dependent manner [3], [4].

Lhp1 is the *S. cerevisiae* homologue of La. The La protein, also known as SS-B, was originally identified as an autoantigen in patients with the rheumatic diseases systemic lupus erythematosus and Sjorgen's syndrome [5], [6]. Since then, homologues have been identified in other eukaryotes [7]–[9]. It is an RNA binding protein with a preference for RNAs with the sequence UUU_OH_ at their 3′ ends [10], [11]. It therefore binds all newly synthesized RNA polymerase III transcripts, as well as other RNAs ending in this sequence. Several studies have revealed that La's major role is to protect the 3′ end of nascent small RNAs from exonuclease digestion. Thus, it plays an important role in a variety of crucial processes, such as stabilization of RNA structure, retention of small RNAs in the nucleus, facilitation of RNP assembly, and accurate tRNA processing [12]. La proteins can essentially be divided into 3 regions. The N terminus contains a La motif, which is also found in several unrelated proteins. This domain appears to be important for RNA binding specificity, even though it is not sufficient to confer RNA binding. The middle portion contains an RNA recognition motif (RRM, also called RNP motif), followed by a more weakly conserved, highly charged C terminus. The C terminal domain of La varies in length and has probably evolved into additional functional domains [12].

Most of the functional roles attributed to La take place in the nucleus, and, as expected, Lhp1 is almost exclusively localized to the nucleus [3], [4]. However, the signal directing nuclear import and the pathway utilized by Lhp1 to enter the nucleus differ from those of the other homologues. Human La contains a classical NLS and is imported into the nucleus via the Kap α/β heterodimer [4], [13]. In contrast, Lhp1 contains a rather large NLS, which overlaps with the RRM, and is imported in a Kap108-dependent manner [3], [4]. Here, in attempting to characterize the Lhp1 NLS in the context of the intact protein, we have found that an Lhp1 mutant containing just 3 point mutations, named RNP1.3, interacts with Kap121 instead of Kap108 and is transported in a Kap121-dependent manner. In contrast, wild type Lhp1 is imported by Kap108 and does not appear to interact with Kap121 *in vivo*. This mutant protein is unstable, specifically interacts with chaperones, and is not fully functional. These results suggest its conformation likely differs from that of wild type Lhp1. Structural studies will be necessary to determine how Kap121 is able to recognize general structural elements.

## Results

### Mutations in full length Lhp1 alter its import pathway

**Figure 1:**
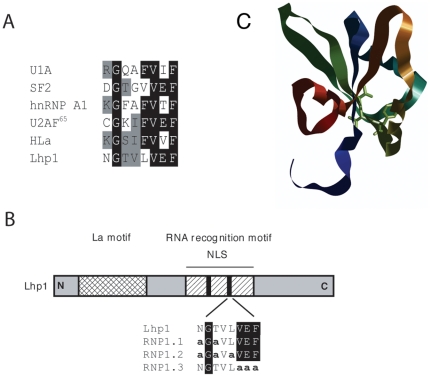
Schematic representation of Lhp1. **A**) Alignment of RNP1 sequences from different proteins. Conserved residues are highlighted **B**) The different domains of Lhp1 are indicated (La motif with a grid, RRM with crossbars, NLS with a solid line). Solid black boxes represent RNPs. Residues altered to alanine in the different mutants are shown in bold lower case. **C**) Ribbon diagram of the human La protein RRM domain (PDB code 1S79) with amino acids mutated to Ala in RRM1.3 shown as tubes.

**Figure 2:**
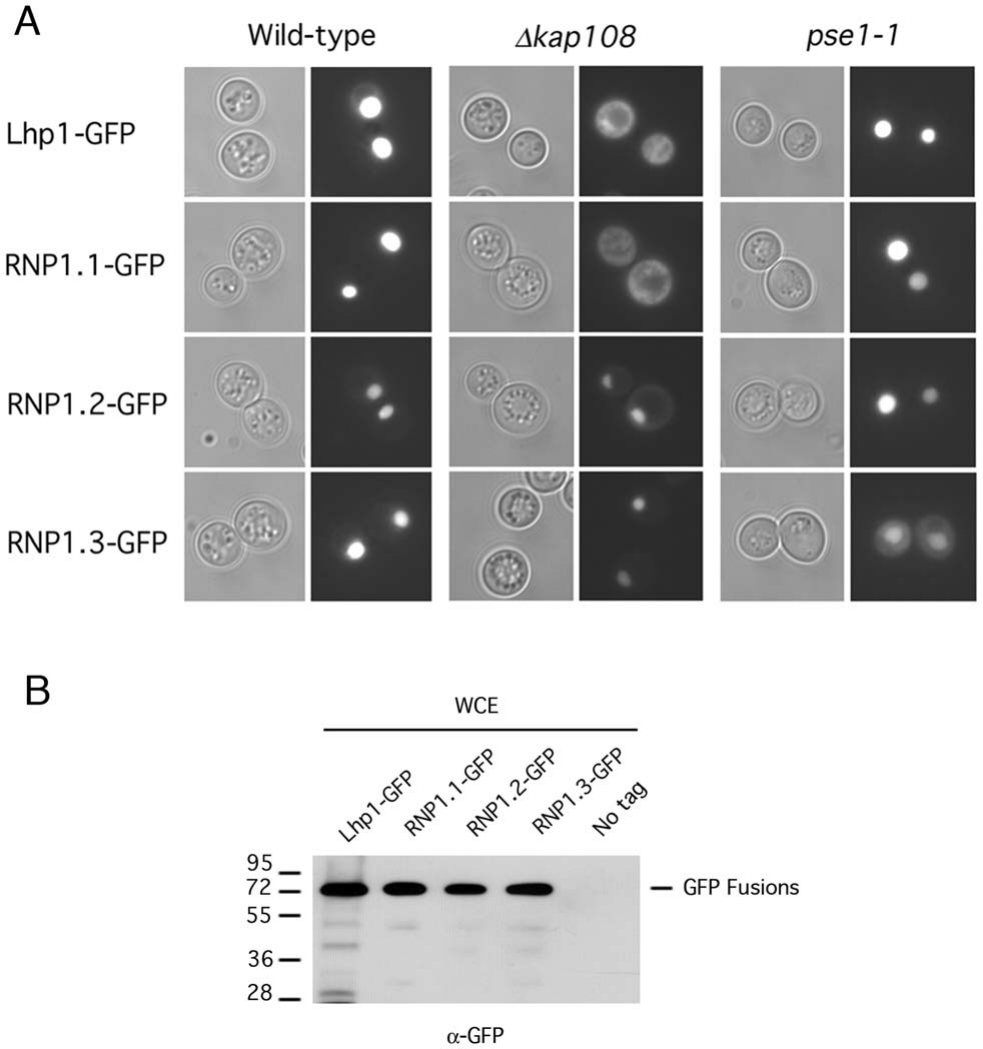
Localization of Lhp1 mutants. **A**) Localization of Lhp1 mutants was assessed in wild type (DF5a), *Δkap108* and the Kap121 mutant strain *pse1-1*, grown at room temperature. GFP fusion proteins were visualized in living cells by virtue of the fluorescent GFP (right panels). Coincident Nomarski (DIC) images are shown (left panels). **B**) Whole cell extracts of the indicated strains were were separated by SDS-PAGE, transferred to nitrocellulose and probed with an anti-GFP antibody. Molecular weights in kDa are shown.

The Lhp1 NLS has been mapped to amino acids 112-224 [4]. However, this region confers a weak nuclear accumulation. It is possible this reflects the existence of additional NLSs. Alternatively, larger domains of Lhp1 could provide a better structural scaffold, resulting in stabilization of the Kap interaction. We therefore sought to characterize this NLS further by mutating several residues in the context of the full-length protein. There are 2 sequences in the RRM that are conserved in proteins containing this RNA-binding motif, RNP1 and RNP2 [14]. We started our approach by altering several residues in one of these, RNP1. In Fig. 1A, RNP1 sequences from several proteins are aligned and conserved residues are highlighted. A schematic representation of the different mutations is shown in Fig. 1B. Mutations were made in residues likely on the protein surface (RNP1.1 and RNP1.2), as well as in conserved residues predicted to fold into the hydrophobic core and therefore with an essential structural role (RNP1.3) [15]. Fig. 1C shows the structure of the human La RRM1 with the conserved residues mutated in RNP1.3 highlighted (VVF). The different mutants were cloned in frame with GFP and their localization was assessed in living cells. As seen in Fig. 2, the localization of the RNP mutants in wild-type cells was indistinguishable from that of Lhp1. Although mutation of these residues had no apparent effect on NLS function in wild type cells, the localization of the mutants in *Δkap108* cells revealed that the import pathway had switched. In this strain, as previously reported, wild type Lhp1 localizes throughout the cell. The RNP1.1 mutant mimics this localization. In contrast, RNP1.2 is only slightly mislocalized to the cytosol, while RNP1.3 retains an almost exclusive nuclear localization. Instead, RNP1.3 is significantly mislocalized in *pse1-1*, a temperature sensitive strain bearing a mutation in Kap121, whereas the localization of the other fusions is unaffected in this strain. The mislocalization of RNP1.3 seen in the *pse1-1* strain is consistent with that seen for other Kap121 substrates in this strain under identical conditions [2], [16], [17]. Western blot of whole cell extracts showed the GFP fusions are not degraded (Fig. 2B), indicating the fluorescence observed corresponds to that of full-length proteins.

### RNP1.3 is unstable and interacts with protein folding chaperones

**Figure 3:**
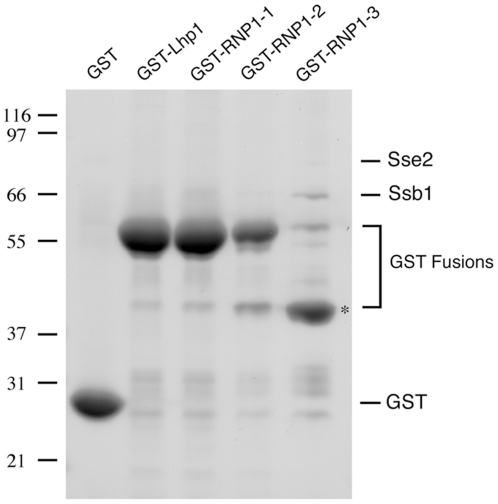
Purification of Lhp1 mutants. Cytosol from an equivalent number of yeast cells expressing GST-tagged Lhp1 mutants was incubated with glutathione sepharose. Bound proteins were washed with transport buffer (TB) and eluted with sample buffer. Proteins of collected fractions were separated by SDS-PAGE and stained with Coomassie Blue. The bands representing GST, the different GST fusions, Ssb1, and Sse2 are indicated. The major degradation product in the preparation, which contains GST, is marked with an asterisk. Molecular weights in kDa are shown.

**Figure 4:**
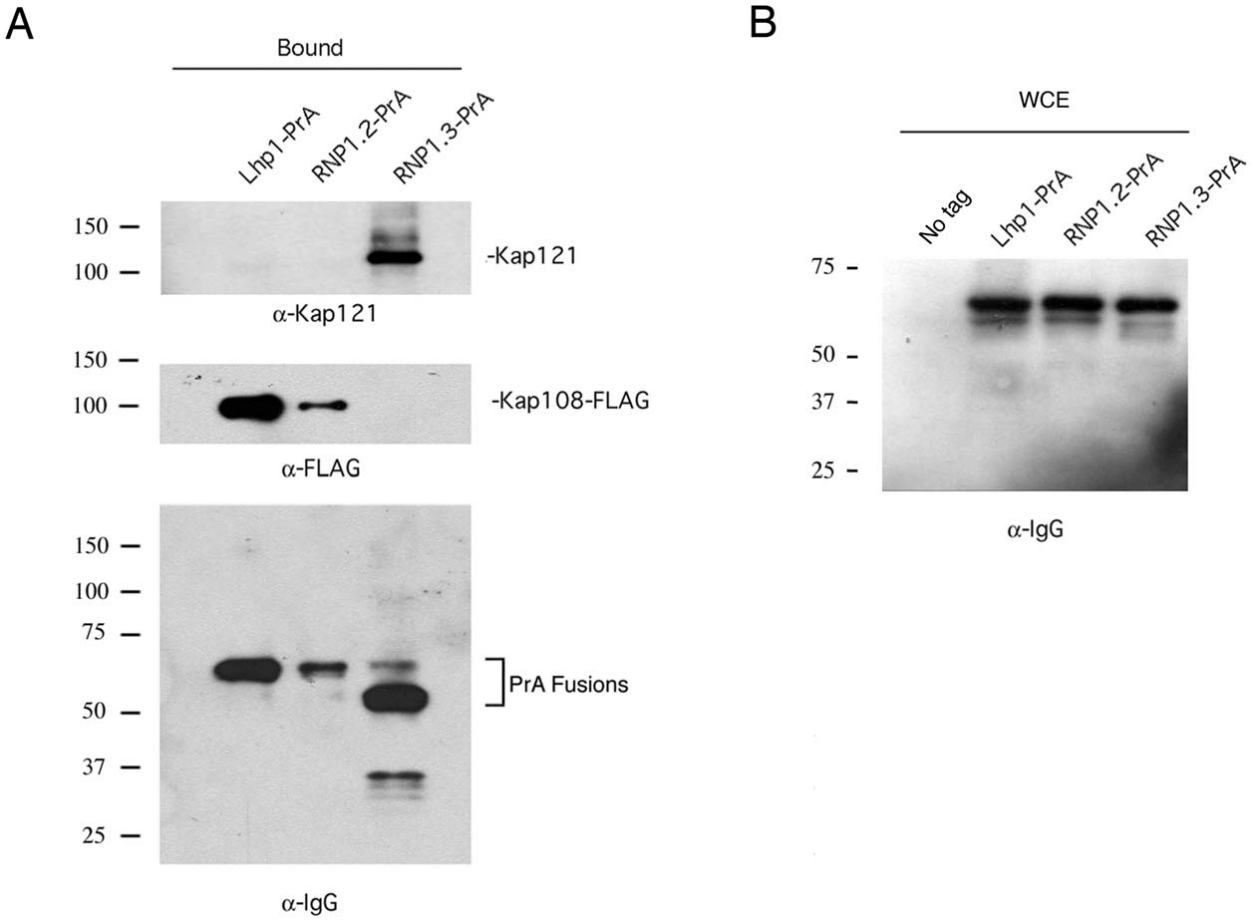
RNP1.3 interacts with Kap121. **A**) Cytosol from cells expressing Lhp1-PrA, RNP1.2-PrA or RNP1.3-PrA was incubated with IgG sepharose. Bound proteins were washed with transport buffer (TB) and eluted with 0.25M and 4.5M MgCl_2_. The fractions containing the 0.25M MgCl_2_ elution are shown. Proteins of collected fractions were separated by SDS-PAGE, transferred to nitrocellulose and probed with an anti-IgG antibody (*lower*), an anti-FLAG antibody (*middle*) or an anti-Kap121 antibody (*upper*). The bands representing the PrA fusions, Kap108-FLAG and Kap121 are indicated. Molecular weights in kDa are shown. **B**) Whole cell extracts of the indicated strains were were separated by SDS-PAGE, transferred to nitrocellulose and probed with an anti-IgG antibody. Molecular weights in kDa are shown.

It is often found that nuclear proteins and their partner Karyopherins form a stable complex in the cytosol prior to their import into the nucleus. We thus sought to determine which proteins interact with the cytosolic pool of the Lhp1 mutant proteins. All mutants were expressed as GST fusions in yeast. The cytosolic fraction of the different strains was incubated with glutathione sepharose, and bound proteins were eluted with sample buffer (Fig. 3). We found that RNP1.3 mainly breaks down into smaller degradation products. Degradation was reduced when the length of the purification procedure was decreased, and mostly full-length protein was detected by Western blot when a cell extract was prepared quickly (not shown, Fig. 4A). This suggests RNP1.3 is unstable out of its normal cellular environment. We also identified 2 proteins interacting with RNP1.3 as Ssb1 and Sse2 (Fig. 3), which have been shown to function as chaperones [18], [19]. This raised the possibility that Kap121 acts as a chaperone for misfolded Lhp1, but we did not detect binding of Kap121 to GST-RNP1.3. However, we also could not detect binding of Kap108 to GST-Lhp1 at the Coomassie level. One possibility was that the GST tag and/or presence of an N-terminal tag interfered with binding. Since Lhp1 tagged with ProteinA (PrA) had been shown to interact with Kap108, we subsequently cloned all mutants in frame with PrA at the C-terminus. However, binding of Kap108 to overexpressed Lhp1-PrA was still not detected by Coomassie blue staining (not shown). It therefore appears that overexpression of Lhp1 from a plasmid interferes with the ability to detect binding to its partner Karyopherin.

### RNP1.3 interacts with Kap121

In view of the above results, we proceeded to genomically insert the RNP1.2 and RNP1.3 mutations in the Lhp1 coding sequence, in frame with Protein A. Lhp1-PrA had previously been described [3]. Since RNP1.1 mimics the behavior of wild-type Lhp1, this mutant was excluded from further analysis. Kap108 was tagged at the C terminus with a FLAG epitope in these strains, to allow identification of Kap108 by Western blot. Proteins interacting with the cytosolic pool of the different fusions were isolated by incubation with IgG sepharose. Bound proteins were eluted with 0.25 M MgCl_2_, which is sufficient to disrupt the Lhp1/Kap108 interaction [3], and the remaining sepharose-bound proteins were eluted with 4.5M MgCl_2_. Collected fractions were precipitated, separated by SDS-PAGE, and transferred to nitrocellulose. The membrane was first probed with an anti-IgG antibody, to detect Protein A fusions, and then with an anti-FLAG antibody to detect FLAG-tagged Kap108. After stripping, the membrane was re-probed with an anti-Kap121 antibody. As seen in Fig. 4, the FLAG tag did not interfere with the Lhp1/Kap108 interaction. We also found that Kap108 binds to RNP1.2, but was not associated with RNP1.3. In contrast, Kap121 interacts with RNP1.3, but not Lhp1 or RNP1.2. Western blot of whole cell extracts showed the degradation pattern of all PrA fusions is identical (Fig. 4B), indicating Kap121 does not interact with a degradation product of RNP1.3 in the cell. We could not detect binding of Kap121 to Lhp1 even in the absence of Kap108 (not shown). These results support the hypothesis that mutation of specific residues in Lhp1 changes its Karyopherin preference.

### RNP1.3 does not maintain wild-type Lhp1 function

**Figure 5:**
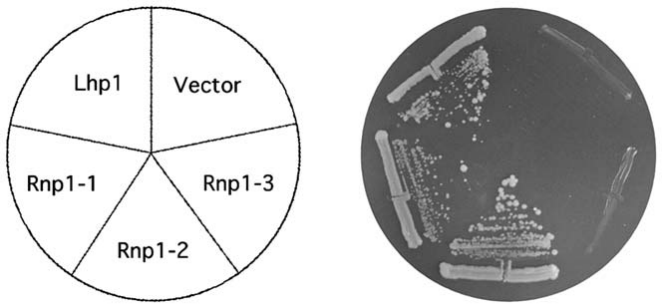
Functional analysis of Lhp1 mutants. Strain CY9T, which depends on a plasmid expressing *LHP1* (*URA3*) for viability, was transformed with pYX242-GFP, Lhp1-GFP, pRNP1.1-GFP, pRNP1.2-GFP and pRNP1.3-GFP. Transformants were streaked on plates containing 5-FOA and allowed to grow for 3–5 days. Results from a representative experiment are shown.

Based on sequence homology to other RRM-containing proteins whose structure was known, we predicted the residues mutated in RNP1.3 were buried in the core of the protein. Thus, it is likely that the entire RNA binding domain of Lhp1 has been disrupted in this mutant. If this is indeed the case, normal function of Lhp1 should be impaired in RNP1.3. We therefore sought to determine whether the mutants we constructed were functional. Lhp1 is not an essential protein, but it becomes indispensable for viability in strains harboring specific additional mutations. For instance, cells where the anticodon stem of tRNA_CGA_ is mutated require Lhp1 [20]. Strains containing both this mutation and an *LHP1* disruption become dependent on a plasmid containing *LHP1* for growth. We therefore transformed this strain with the different RNP-GFP fusions in order to determine whether these could functionally substitute for Lhp1. The original plasmid containing *LHP1* carries the *URA3* gene. Since 5-fluoro-orotic acid (5-FOA) is toxic for cells expressing *URA3*, it was possible to counter-select this plasmid. As expected, we found that GFP alone cannot substitute for Lhp1 (Fig. 5). In addition, the GFP tag does not interfere with Lhp1's normal function. RNP1.1 and RNP1.2 were also functional but, in contrast, RNP1.3 could not substitute for Lhp1 in this assay (Fig. 5). This indicates that this mutant cannot perform at least one of Lhp1's original functions.

## Discussion

Import of Lhp1 into the nucleus is a Kap108-dependent process. We have found that mutation of 3 residues in the Lhp1 RNP1 motif results in a switch of the main carrier utilized for import from Kap108 to Kap121. This mutant (RNP1.3) interacts with Kap121 but not Kap108 *in vivo*, and is mislocalized to the cytoplasm in a strain defective in Kap121 function, but not in a Kap108 deficient strain. In contrast, we could not find any evidence implicating Kap121 in the import of wild type Lhp1 into the nucleus, even as a backup Kap. These results indicate that RNP1.3 is a specific cargo for Kap121, whereas wild type Lhp1 is not.

It seems evident that the switch in the Lhp1 import pathway is not a general effect of mutations in RNP1. We could not detect binding of Kap121 to RNP1.2, with 3 different residues in RNP1 altered, nor did we observe mislocalization of this mutant in *pse1-1*. However, our results indicate that the RNP1.3 structure differs from that of RNP1.2 and wild-type Lhp1. RNP1.3 interacts with protein folding chaperones, suggesting that it is not properly folded, and it is unstable outside the cellular environment. In RRM-containing proteins whose structure is known, the conserved RNP1 residues altered in RNP1.3 are part of the hydrophobic core of the RRM motif [15]. Thus, these mutations likely caused more than a slight structural change in this domain and disrupted its structural integrity. This is consistent with the finding that RNP1.3 does not complement Lhp1 in a functional assay and it interacts with chaperones. Therefore, the change in import pathways likely results from destabilization of the Lhp1 structure. In support of this, Lhp1 mutants where 8 residues in either RNP1, RNP2, or both RNPs are replaced with alanine are mislocalized in *pse1-1* but not *Δkap108* or different Kap mutants (not shown).

Both RNP1 and RNP2 form central strands in the β sheet that is characteristic of the RRM motif. Mutation of all residues in either of these conserved sequences undoubtedly prevents correct folding of this domain. At the present, it is unclear how this would result in a higher affinity of Kap121 for Lhp1. One possibility is that Kap121 specifically recognizes the overall surface properties of certain proteins, rather than particular sequence or structural elements. This is reminiscent of the way chaperones interact with their substrates. Chaperones recognize peptides containing hydrophobic residues and/or polypeptide backbone regions, both structural features of unfolded or partially unfolded proteins [21], [22]. Such binding specificity allows them to distinguish between native and non-native conformations, so that they can assist in protein folding. Kap121 has been shown to function as a backup Kap in several pathways [16], [23], [24], but it also displays a high degree of specificity towards its main transport substrates [25], [26]. It is therefore likely that Kap121 binding specificity and cargo selectivity are determined by several mechanisms.

As mentioned above, RNP1.3 is likely to contain a misfolded RRM. It was therefore surprising that this protein is still targeted to the nucleus. Why would a Kap transport a misfolded protein into the nucleus? Nuclear proteins are synthesized in the cytoplasm, where they are not likely to meet their binding partners. It is therefore possible that Kaps associate with proteins for which they have affinity in the cytoplasm and maintain them in a protected state until they can deliver them to their proper location in the nucleus. In this sense, they would function as chaperones. It has been suggested that Kaps function as chaperones for exposed basic domains, as the presence of several Karyopherins suppressed the aggregation of their basic import cargoes in polyanionic environments [27]. The notion that Kaps recognize basic residues seems to be favored in the literature. Yet, this has only been clearly demonstrated for Kap α, which recognizes proteins containing classical NLSs [28]. It seems likely that individual Kaps have a strong preference for distinct features. For instance, Crm1, the best-studied export factor, binds to leucine-rich sequences [28]. In addition, Kap142 has been shown to export phosphorylated cargoes [1]. On the other hand, the M9 NLS recognized by Kapβ2 is rich in glycine residues [29], [30]. Nonetheless, since only a limited number of NLSs recognized by each Kap have been mapped, it is not yet possible to generalize a mechanism for cargo recognition by distinct Karyopherins. It has been suggested that several proteins with a chromatin-related function possess a consensus basic sequence for import by Kap121 and Kap123 [31]. However, it has also been reported that Kap121 recognizes arginine and glycine-rich NLSs in addition to lysine-rich NLSs [32]. Our results show that Kap121 specifically recognizes a protein whose core structure has been disrupted. Thus, it is possible that Kap121 also has affinity for newly exposed hydrophobic residues.

It had been shown that the nuclear import mechanism of *S. cerevisiae* La differs from that of homologues in higher eukaryotes, whose transport is mediated by the Kap α/β heterodimer. In contrast, Lhp1 is imported via Kap108. Rosenblum and coworkers proposed that evolution of La to attain additional functionality might have included a change in the NLS [4]. In order to change the region of La that functions as an NLS and the Kap used, a common ancestor of yeast and human La would have had to undergo mutation while retaining nuclear localization. We have shown that mutation of merely 3 residues in Lhp1 changes its import pathway and, accordingly, its affinity for Karyopherins. This finding provides an example of how Karyopherins may provide buffering from genetic variation by adjusting transport pathways. It is generally accepted that chaperones control the expression of genetic variation as phenotypic variation by assisting in the folding of structurally unstable mutant proteins [33]. So, while deleterious effects of particular alleles are masked, functionally advantageous mutations can arise. In parallel, the Kap network could allow proteins to acquire mutations in a region that disrupts a localization signal for one pathway without compromising their proper localization.

## Materials and Methods

### Plasmids

PCR fragments containing mutations in *LHP1* were created by the overlap extension method and cloned into pYX242-GFP [4] by homologous recombination to generate pRNP1.1-GFP, pRNP1.2-GFP, and pRNP1.3-GFP. Oligonucleotides that introduced alanine codons in place of wild-type sequences were used. Lhp1-GFP was described previously [4]. LHP1, RNP1.1, RNP1.2, and RNP1.3 were PCR-amplified from Lhp1-GFP, pRNP1.1-GFP, pRNP1.2-GFP, and pRNP1.3-GFP and ligated into the bacterial expression vector pGEX4T1 using primer-encoded BamHI and XhoI sites, resulting in N-terminal fusions with GST. GFP was replaced with the coding sequence of four and a half IgG binding repeats of *S. aureus* protein A in Lhp1-GFP, pRNP1.1-GFP, pRNP1.2-GFP, and pRNP1.3-GFP by homologous recombination to generate pRNP1.1-PrA, pRNP1.2-PrA, and pRNP1.3-PrA. All constructs were verified by sequencing analysis. XL1-blue (Stratagene, La Jolla, CA) was used for general cloning and expression purposes. Bacterial cells were transformed by heat shock.

### Yeast strains

The procedures for *S. cerevisiae* manipulation were as described [34]. *pse1-1* [35], *Δkap108* and Lhp1-PrA [3], and CY9 [20] have been described. The auxotrophy marker of CY9 was switched from LEU to TRP by transformation with a fragment from pLT11 [36] to generate CY9T. A strain expressing RNP1.2-PrA was obtained using the method *delitto perfetto* [37]. Briefly, Lhp1-PrA was streaked on plates containing 5-FOA to induce mutations in the *URA3* gene. A URA^-^ colony was selected and nucleotides 301 to 588 of *LHP1* deleted in this strain by replacement with *KAN* and *URA3* amplified from pCORE [37], resulting in *LHP1*::CORE-PrA. A fragment of *LHP1* containing the RNP1.2 mutation was obtained by digestion of pRNP1.2-GFP with EcoRI and transformed into *LHP1*::CORE-PrA. Cells were plated on YPD, grown overnight at 30°C, and replica plated onto plates containing 5-FOA to select for loss of *URA3*. Cells were allowed to grow an additional 2 days and resulting colonies were tested for the loss of the KAN marker. Proper integration of RNP1.2 was assessed by PCR, western blot, and sequencing analysis. We could not obtain RNP1.3-PrA using this method and therefore used a different strategy: Nucleotides 301 to 588 of *LHP1* were deleted in DF5a by replacement with *KAN* and *URA3* amplified from pCORE to obtain *LHP1*::CORE. A PCR fragment containing RNP1.3-PrA and the *HIS5* gene from *S. pombe* (RNP1.3-PrA/*HIS5*) was created by overlap extension. Templates for this reaction were RNP1.3-PrA amplified from pRNP1.3-PrA and the *HIS5* gene amplified from pBxA [38] containing the last 40 nucleotides of PrA at the 5′ end and the 60 nucleotides immediately downstream of the *LHP1* stop codon at the 3′ end. Transformation of RNP1.3-PrA/*HIS5* into *LHP1*::CORE yielded RNP1.3-PrA. Proper integration of RNP1.3 was confirmed by PCR, western blot, and sequencing analysis. RNP1.2-PrA and RNP1.3-PrA expressing Kap108-FLAG were obtained by integrative transformation. The cassette used contained the FLAG epitope coding sequence and the *TRP* gene flanked by the 60 nucleotides immediately upstream and 60 nucleotides directly downstream of the *KAP108* stop codon, and was PCR amplified from pESC-TRP (Stratagene, La Jolla, CA). Proper integration was confirmed by PCR and western blot.

### Cell Fractionation and Immunoisolation

Post-nuclear, post-ribosomal cytosol was prepared from Protein A or GST tagged strains grown to an OD_600_ of 1.8 as described [39]. Tagged proteins and proteins with which they interact were isolated by incubation of cytosol with IgG sepharose or glutathione sepharose at 4°C. After washing with TB (20 mM HEPES pH 7.5, 110 mM KOAc, 2 mM MgCl_2_, 0.1% Tween-20), proteins were eluted with either sample buffer or a step gradient of MgCl_2_ followed by concentration using methanol-chloroform precipitation. Fractions were analyzed by SDS-PAGE, and gels were stained with Commassie blue. Proteins of interest were excised, digested and analyzed by MALDI-TOF mass spectrometry [40]. Alternatively, gels were transferred to nitrocellulose.

### Western blotting

Western blotting procedures and subsequent chemiluminescence detection were as described by the manufacturer (Pierce). The primary antibodies used were anti-FLAG (Sigma#F-3165), anti-IgG (Sigma#A-9044), anti-GFP (Roche#11814460001), and anti-Kap121 (a generous gift from Dr. Rick Wozniak, University of Alberta).

### Fluorescent microscopy

GFP was visualized after overnight growth of yeast strains in medium or on agar selective for the respective plasmid. 5 µl of culture or one colony resuspended in 5 µl of water was placed on a slide. All images were viewed with a 63x oil objective on a Zeiss Axiophot microscope. Images were collected with a Hamamatsu video imaging system using Openlab software and transferred to Adobe Photoshop.
